# Tissue‐Specific Effects of Dietary Protein on Cellular Senescence Are Mediated by Branched‐Chain Amino Acids

**DOI:** 10.1111/acel.70176

**Published:** 2025-07-28

**Authors:** Mariah F. Calubag, Ismail Ademi, Isaac D. Grunow, Lucia E. Breuer, Reji Babygirija, Penelope Lialios, Sandra M. Le, Michelle M. Sonsalla, Julia A. Illiano, Bailey A. Knopf, Fan Xiao, Dennis Minton, Adam R. Konopka, David A. Harris, Dudley W. Lamming

**Affiliations:** ^1^ Department of Medicine University of Wisconsin‐Madison Madison Wisconsin USA; ^2^ William S. Middleton Memorial Veterans Hospital Madison Wisconsin USA; ^3^ Cell and Molecular Biology Graduate Program University of Wisconsin‐Madison Madison Wisconsin USA; ^4^ Comparative Biomedical Sciences Graduate Program University of Wisconsin‐Madison Madison Wisconsin USA; ^5^ Department of Surgery, Wisconsin Surgical Laboratory in Metabolism University of Wisconsin‐Madison Madison Wisconsin USA; ^6^ Nutrition and Metabolism Graduate Program University of Wisconsin‐Madison Madison Wisconsin USA; ^7^ University of Wisconsin Comprehensive Diabetes Center, University of Wisconsin‐Madison Madison Wisconsin USA; ^8^ Wisconsin Nathan Shock Center of Excellence in the Basic Biology of Aging Madison Wisconsin USA; ^9^ University of Wisconsin Carbone Comprehensive Cancer Center, University of Wisconsin‐Madison Madison Wisconsin USA

## Abstract

Dietary protein is a key regulator of healthy aging in both mice and humans. In mice, reducing dietary levels of the branched‐chain amino acids (BCAAs) recapitulates many of the benefits of a low protein diet; BCAA‐restricted diets extend lifespan, reduce frailty, and improve metabolic health, while BCAA supplementation shortens lifespan, promotes obesity, and impairs glycemic control. Recently, high protein diets have been shown to promote cellular senescence, a hallmark of aging implicated in many age‐related diseases, in the liver of mice. Here, we test the hypothesis that the effects of high protein diets on metabolic health and on cellular senescence are mediated by BCAAs. We find that reducing dietary levels of BCAAs protects male mice from the negative metabolic consequences of both normal and high protein diets. Further, we identify tissue‐specific effects of BCAAs on cellular senescence, with restriction of all three BCAAs—but not individual BCAAs—protecting from hepatic cellular senescence while potentiating cellular senescence in white adipose tissue. We also find that these effects are sex‐specific. We find that the effects of BCAAs on hepatic cellular senescence are cell‐autonomous, with lower levels of BCAAs protecting cultured cells from antimycin‐A induced senescence. Our results demonstrate a direct effect of a specific dietary component on a hallmark of aging and suggest that cellular senescence may be highly susceptible to dietary interventions.

## Graphical abstract

Restriction of dietary BCAAs protects male mice from the metabolic consequences of both normal‐ and high‐protein diets. BCAA restriction also protects from hepatic cellular senescence in vivo and in vitro, especially in the context of mitochondrial stress.

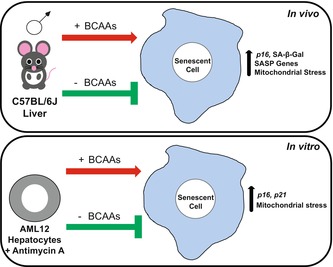

## 1 Introduction

As the global population ages, there is growing interest—and urgency—to identify effective and affordable interventions to promote healthy aging (The Lancet Healthy 2022). One of the 12 hallmarks of aging (Lopez‐Otin et al. 2023), cellular senescence, has attracted significant attention as a potential target for therapeutic intervention as the accumulation of senescent cells and their associated mix of cytokines, chemokines, and other factors, collectively known as the senescence‐associated secretory phenotype (SASP), is linked to a broad array of age‐related diseases (D. G. Burton 2009; Burton and Faragher 2018; Calubag et al. 2024; Campisi and d'Adda di Fagagna 2007; Childs et al. 2015; Coppe et al. 2010; Gavina et al. 2022; Kawai et al. 2021; Lopez‐Otin et al. 2013; Munoz‐Espin and Serrano 2014; Ovadya and Krizhanovsky 2014; Palmer, Jensen, et al. 2022; Palmer, Tchkonia, and Kirkland 2022; Roberto et al. 2015; Santos and Sinha 2021; van Deursen 2014).

While dietary interventions are appealing, calorie restriction (CR), the gold standard for aging interventions, is difficult for many (Mihaylova et al. 2023). Recently, dietary composition—especially dietary protein—has been shown to have strong effects on healthy aging (Green, Lamming, and Fontana 2022). While more protein is typically thought of as beneficial, higher protein intake is associated with increased mortality and age‐related diseases in humans (Levine et al. 2014; Sluijs et al. 2010). Conversely, protein restriction (PR) promotes metabolic health in both humans and rodents and extends rodent lifespan (Ferraz‐ Bannitz et al. 2022; Fontana et al. 2016; Hill et al. 2022; Maida et al. 2016; Richardson et al. 2021; Ross 1961; Solon‐Biet et al. 2014). It was recently shown that as dietary protein increases, hepatic senescence increases (Nehme et al. 2021).

While the mechanism by which dietary protein promotes senescence is unknown, many of the metabolic benefits of PR are mediated by reduced dietary levels of the three branched‐chain amino acids (BCAAs; leucine, isoleucine, and valine). BCAAs are powerful regulators of healthy aging, with restriction of all three BCAAs or isoleucine alone improving metabolic health, reducing frailty, and extending lifespan (Cummings et al. 2018; Fontana et al. 2016; Richardson et al. 2021). In contrast, BCAA supplementation promotes obesity and insulin resistance and reduces lifespan (Newgard et al. 2009; Solon‐Biet et al. 2019). An accumulating set of data suggests that BCAAs promote senescence, with BCAA supplementation promoting senescence in cell culture experiments (Nakano et al. 2013) as well as in vivo in mice (Mu et al. 2018). Genetic manipulation of BCAA catabolism in cells and mice likewise is consistent with BCAAs promoting senescence (Han et al. 2023; Liang et al. 2023).

Thus, it is possible that dietary protein promotes senescence via the BCAAs. If this hypothesis is correct, several molecular mechanisms could contribute to BCAA‐mediated senescence. Protein in general, and the BCAAs in particular, are potent agonists of the mechanistic Target of Rapamycin Complex 1 (mTORC1), a highly conserved protein kinase that, when inhibited, extends the lifespan of mice (Mannick and Lamming 2023; Simcox and Lamming 2022). Protein restriction and BCAA restriction both reduce mTORC1 activity in mice (Lamming et al. 2015; Solon‐Biet et al. 2014), and mTORC1 promotes the SASP; rapamycin, which extends mouse lifespan by inhibiting mTORC1, inhibits the SASP (Laberge et al. 2015). Additionally, both PR and BCAA restriction induce fibroblast growth factor 21 (FGF21), a hormone that extends lifespan when overexpressed and regulates mTORC1 in multiple tissues (Fontana et al. 2016; Gong et al. 2016; Laeger et al. 2016; Minard et al. 2016; Zhang et al. 2012). In vitro, knockdown of FGF21 promotes the accumulation of senescent cells (Li et al. 2019) whereas FGF21 overexpression and administration have been shown to protect against senescence (Li et al. 2019; Lu et al. 2021).

In this study, we tested the hypothesis that the effects of dietary protein on senescence are mediated by the BCAAs. We found that restriction of the dietary levels of BCAAs protects male mice from the negative metabolic consequences of both normal and high protein diets, and that the dietary levels of BCAAs—but not protein—are associated with hepatic senescence and SASP gene expression.

Further, we discovered tissue‐specific effects of BCAAs on cellular senescence, with restriction of all three BCAAs—but not individual BCAAs—protecting from hepatic cellular senescence while potentiating cellular senescence in white adipose tissue. Despite previous literature, we found that this tissue‐specific effect is independent of the FGF21/mTORC1 axis, but rather mediated by an effect of BCAAs on mitochondrial function. We found that the effect of BCAAs on hepatic cellular senescence is cell‐autonomous, with lower levels of BCAAs protecting cultured hepatocytes from antimycin‐A induced senescence. We conclude that dietary BCAA restriction can promote metabolic health even in the context of higher protein diets, and that BCAAs are a dietary component that affects senescent cell accumulation in a tissue‐specific manner.

## 2 Materials and Methods

### 2.1 Animal Care, Housing, and Diet

All procedures were performed in conformance with institutional guidelines and were approved by the Institutional Animal Care and Use Committee (IACUC) of the William S. Middleton Memorial Veterans Hospital and the University of Wisconsin‐Madison IACUC. Male C57BL/6J mice were purchased from The Jackson Laboratory (Bar Harbor, ME, USA) at 11 weeks of age. p16‐3MR reporter mice on the C57BL/6J background, in which the p16^INK4a^ promoter drives expression of the 3MR fusion protein, (Demaria et al. 2014) were received at 13 weeks of age from the Harris and Ricke labs at UW‐Madison, which were generously gifted to them by Dr. Judith Campisi. All mice were acclimated to the animal research facility for at least 1 week before entering studies. All animals were housed in static microisolator cages in a specific pathogen‐free mouse facility with a 12:12 h light–dark cycle, maintained at approximately 22°C.

Mice were fed amino acid‐defined diets with varying levels of protein and BCAAs (full diet compositions are provided in Table S1; Inotiv, Madison, WI, USA). Diets were started at either 12 weeks of age or 16 months of age in the C57BL/6J mice, or at 14 weeks of age in the p16‐3MR mice, and continued for at least 16 weeks. 8‐week‐old male C57BL/6J mice were placed on a high‐fat high‐sucrose western diet (WD; TD.160186) or a WD with restricted BCAAs (WD‐BR; TD.160188) for 45 weeks.

### 2.2 Metabolic Phenotyping

Glucose, insulin, and alanine tolerance tests were performed by fasting all mice for 4 h or overnight (~16 h) and then injecting either glucose (1 g/kg), insulin (0.75 U/kg) or pyruvate (2 g/kg) intraperitoneally (Bellantuono et al. 2020; Yu et al. 2019).

Blood glucose levels were determined at the indicated times using a Bayer Contour blood glucose meter (Bayer, Leverkusen, Germany) and test strips. Mouse body composition was determined using an EchoMRI Body Composition Analyzer. For the assay of multiple metabolic parameters (O_2_, CO_2_, food consumption, and activity tracking), mice were acclimatized to housing in a Columbus Instruments Oxymax/CLAMS‐HC metabolic chamber system for approximately 24 h, and data from a continuous 24‐h period was then recorded and analyzed.

### 2.3 In Vivo Bioluminescence

In vivo luminescence assessment was performed by the Small Animal Imaging & Radiotherapy Facility (SAIRF). p16‐3MR reporter mice were injected intraperitoneally with 15 μg of RediJect Coelenterazine h Bioluminescent Substrate (Fischer Scientific; Catalog no. 50‐209‐9325). 15 min later, the mice were anesthetized with isoflurane, and luminescence was measured with a Revvity IVIS Spectrum in vivo imaging system (Revvity Health Sciences; 1 min medium binning).

### 2.4 Collection of Tissues for Molecular and Histological Analysis

Mice were euthanized in the fasted or fed state at the indicated age. Mice euthanized in the fed state were fasted overnight starting the day prior to sacrifice; in the morning, mice were refed for 3 h and then sacrificed 3 h later. Following blood collection via submandibular bleeding, mice were euthanized by cervical dislocation, and tissues were rapidly collected, weighed, and snap frozen in liquid nitrogen. A portion of the liver was fixed in 10% formalin for 4 h, transferred to sucrose for 24 h, and then embedded in Tissue‐Tek Optimal Cutting Temperature (OCT) compound, and then cryosectioned and stained for SA‐β‐gal activity and immunofluorescence staining for gamma‐H2AX. Images of the liver were taken using an EVOS microscope (Thermo Fisher Scientific Inc., Waltham, MA, USA) at a magnification of 40X as previously described (Calubag et al. 2022; Cummings et al. 2018; Yu et al. 2021). For quantification, three independent fields were obtained for each tissue from each mouse and quantified using ImageJ (NIH, Bethesda, MD, USA).

### 2.5 Cell Culture and Treatment

AML12 cells were obtained from American Type Culture Collection (ATCC). AML12 cells were cultured in Gibco Dulbecco's modified Eagle's medium with F12 (DMEM/F12) without Amino Acids, Glucose, L‐Glutamine, Sodium Bicarbonate, HEPES, Sodium Pyruvate, Hypoxanthine, Thymidine, Phenol Red (Powder) (D9807‐10; US Biological Life Sciences, Waltham, MA, USA) supplemented with 10% fetal bovine serum (FBS) (12306C; Life Technologies, Carlsbad, CA, USA) and 1% Penicillin/Streptomycin (15140122; Gibco, Billings, MT, USA). Amino acids and glucose were added to create custom DMEM lacking BCAAs. See Table S5 for amino acid and complete cell culture composition and catalog numbers. Senescence was induced in AML12 cells by treating them with 1 μM Antimycin A (A8674‐25MG; Sigma, St. Louis, MO, USA) for 14–21 days prior to collection (concentration chosen based on (Hytti et al. 2019; Stöckl et al. 2006; Wiley et al. 2016)).

### 2.6 Quantitative Real‐Time PCR

RNA was extracted from liver or inguinal white adipose tissue (iWAT) using TRI Reagent according to the manufacturer's protocol (Sigma‐Aldrich). The concentration and purity of RNA were determined by absorbance at 260/280 nm using Nanodrop (Thermo Fisher Scientific). 1 μg of RNA was used to generate cDNA (Superscript III; Invitrogen, Carlsbad, CA, USA). Oligo dT primers and primers for real‐time PCR were obtained from Integrated DNA Technologies (IDT, Coralville, IA, USA); sequences are in Table S2. Reactions were run on a StepOne Plus machine (Applied Biosystems, Foster City, CA, USA) with Sybr Green PCR Master Mix (Invitrogen). If our technical replicates have a difference in Ct larger than 1, we excluded the sample entirely and did not do further analysis. Actin was used to normalize the results from gene‐specific reactions.

### 2.7 Immunoblotting

Animals used for Western blotting were sacrificed following an overnight fast and 3‐h refeed. Tissue samples from muscle were lysed in cold RIPA buffer supplemented with phosphatase inhibitor and protease inhibitor cocktail tablets (Thermo Fisher Scientific, Waltham, MA, USA) as previously described (Pak et al. 2021; Richardson et al. 2021) using a FastPrep 24 (M.P. Biomedicals, Santa Ana, CA, USA) with screw cap microcentrifuge tubes (822‐S) from (Dot Scientific, Burton, MI) and ceramic oxide bulk beads (10158–552) from VWR (Radnor, PA, USA). Protein lysates were then centrifuged at 13,300 rpm for 10 min, and the supernatant was collected. Protein concentration was determined by Bradford (Pierce Biotechnology, Waltham, MA, USA). 20 μg protein was separated by SDS–PAGE (sodium dodecyl sulfate–polyacrylamide gel electrophoresis) on 16% resolving gels (Thermo Fisher Scientific, Waltham, MA, USA) and transferred to PVDF membrane (EMD Millipore, Burlington, MA, USA). pT389‐S6K1 (9234), S6K1 (9202), pS240/244‐S6 (2215), S6 (2217), p‐Thr37/46 4E‐BP1 (2855), and 4E‐BP1 (9644) were purchased from Cell Signaling Technologies (CST, Danvers, MA, USA) and used at a dilution of 1:1000. Imaging was performed using a Bio‐Rad Chemidoc MP imaging station (Bio‐Rad, Hercules, CA, USA). Quantification was performed by densitometry using NIH ImageJ software.

### 2.8 Senescence‐Associated β‐Galactosidase Staining

Enhanced lysosomal biogenesis, a common feature of cellular senescence, can be detected by measuring β‐galactosidase (lysosomal hydrolase) activity at pH 6.0 (Lee et al. 2006). This senescence‐associated β‐galactosidase (SA‐β‐Gal) activity appears to be restricted to senescent cells at a low pH (Dimri et al. 1995; Zhao et al. 2017). SA‐β‐Gal activity is shared by almost all senescent cells, but particular care should be taken when detecting this in cultured cells because high confluency and contact inhibition can increase SA‐β‐gal activity (Dimri et al. 1995; Melk et al. 2004). Fresh tissue from mice was collected and fixed in 10% neutral buffered formalin (NBF) on ice for 3–4 h. Tissues were then transferred to 30% sucrose at 4°C for 24 h before being embedded in O.C.T. compound in a cryomold and stored at −80°C. Prior to cryosectioning, tissues were equilibrated at −20°C and then cryosectioned into 5–7 μm sections before being attached to Superfrost Plus slides. Fresh SA‐β‐gal staining solution at a pH of 6 was prepared as previously described (Yousefzadeh et al. 2020). Tissue slides were stained in SA‐β‐gal staining solution for 18–24 h at 37°C in a non‐CO2 incubator before being rinsed three times with PBS. To prevent crystal formation, a parafilm Coplin jar was used to prevent evaporative loss of staining solution. Stained sections were imaged using the EVOS microscope (Thermo Fisher Scientific Inc., Waltham, MA, USA) at a magnification of 40X as previously described. The percent of SA‐β‐Gal‐positive area for each sample will be quantified using ImageJ.

### 2.9 Immunofluorescence Staining

The number of gamma‐H2AX foci increases in damaged and senescent cells (Bernadotte et al. 2016), making it a useful marker for DNA damage. We used the same tissues prepared for SA‐β‐Gal staining, and prior to cryosectioning, tissues were equilibrated at −20°C and then cryosectioned into 5–7 μm sections before being attached to Superfrost Plus slides. After blocking the slides with 1X PBS + 5% normal serum +0.3% Triton X‐100 for 1 h, slides were incubated with the primary antibody (p‐H2AX, Ser139, 9718, CST; 1:800 dilution) at 4°C overnight. Slides were further incubated with secondary antibody (Alexafluor 488 Conjugate, 4412: 1:200 dilution) at room temperature for 2 h. After rinsing slides with PBS three times, slides were mounted with Fluoro‐Gel II with DAPI (Electron Microscopy Scientific, Cat#17985–50). Stained sections were imaged using the EVOS microscope (Thermo Fisher Scientific Inc., Waltham, MA, USA) at a magnification of 40X as previously described. The percent of gamma‐H2AX‐positive area for each sample was quantified using ImageJ.

### 2.10 ELISA Assays and Kits

Blood plasma for FGF21 was obtained at Week 15 in the fasted state and from blood collected at the time of euthanasia in the refed state. Blood FGF21 levels were assayed by a mouse/rat FGF‐21 Quantikine ELISA kit (MF2100) from R&D Systems (Minneapolis, MN, USA).

### 2.11 Statistics

Data are presented as the mean ± SEM unless otherwise specified. Statistical analyses were performed using one‐way or two‐way ANOVA followed by Tukey–Kramer post hoc test, as specified in the figure legends. Outliers were excluded using the Robust Regression Outlier Test (ROUT) in Graphpad Prism (v10), *Q* = 1%, and are indicated by an asterik(*) and blue colored font in the Source Data. Other statistical details are available in the figure legends. Energy expenditure differences were detected using analysis of covariance (ANCOVA). ANCOVA analysis assumes a linear relationship between the variables and their covariates. If the slope is equal between groups, then the regression lines are parallel, and elevation is then tested to determine any differences (i.e., if slopes are statistically significantly different, elevation will not be determined). In all figures, *n* represents the number of biologically independent animals. Sample sizes were chosen based on our previously published experimental results with the effects of dietary interventions (Cummings et al. 2018; Fontana et al. 2016; Yu et al. 2021, 2019, 2018). Data distribution was assumed to be normal, but this was not formally tested.

### 2.12 Randomization

All studies were performed on animals or on tissues collected from animals. Young animals of each sex and strain were randomized into groups of equivalent weight, housed 2–3 animals per cage, before the beginning of the in vivo studies. Aged animals were randomized into groups of equivalent weight, housed 2–3 animals per cage, when possible.

## 3 Results

### 3.1 Restricting BCAAs Improves the Metabolic Health and Increases Energy Expenditure of Mice Eating Normal and High Protein Diets

We began our study by designing a series of diets in which the total amount of calories derived from amino acids (AAs) was either 7% (LP, Low Protein), 21% (CP, Control Protein), or 36% (HP, High Protein). These diets were isocaloric. Dietary fat was held constant (19%) and the levels of carbohydrates adjusted in order to maintain equivalent caloric density. We further designed CP and HP diets in which the levels of all three BCAAs were reduced to the level of BCAAs found in the LP diet (CP‐BR and HP‐BR, respectively); in these diets, the reduction in BCAAs was balanced by a proportional increase in non‐essential AAs, keeping the percentage of calories derived from AAs constant. The full composition of these diets is summarized in Table S1.

**FIGURE 1:**
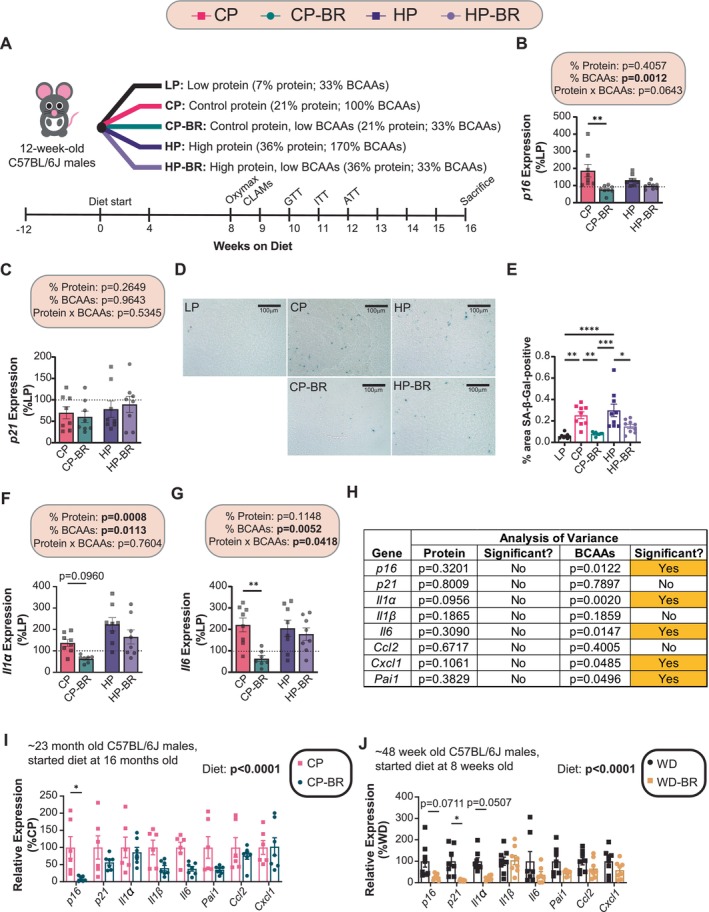
Diets low in BCAAs protect C57BL/6J male mice from the accumulation of senescent cells in the liver. (A) Experimental design. (B, C) Hepatic mRNA expression of *p16* and *p21* in mice fed the indicated diets. (D, E) Hepatic SA‐β‐Gal staining at 40X magnification (scale bar = 100 μm) (D) with quantification of SA‐βGal‐positive cells (E). (F, G) Hepatic mRNA expression of *Il1α* and *Il6* in mice fed the indicated diets. (H) Multiple linear regression (MLR) analysis to determine contribution of protein versus BCAAs to hepatic senescence gene expression (*p16, p21, Il1α, Il1b, Il6, Ccl2, Cxcl1* and *Pai1*). (I) Hepatic mRNA expression of senescence genes in male C57BL/6J mice fed the CP and CP‐BR diets for ~7 months (from 16 to 23 months of age). (J) Hepatic mRNA expression of senescence genes in ~48 week old male mice after *a* ~ 42 weeks consuming a WD‐BR diet. (B, C, F, G) *n* = 7–8 mice/group (100% = average expression of the indicated gene in the liver of LP‐fed mice). The overall effect of protein, BCAAs, and the interaction represent the *p*‐value from a two‐way ANOVA; *p < 0.05, **p < 0.01, Sidak's test post 2‐way ANOVA. (E) *n* = 8–9 mice/group; *p < 0.05, **p < 0.01, ***p < 0.001, ****p < 0.0001. Tukey test post ANOVA. (H) Statistics for the *p*‐value are from a MLR analysis to determine the contribution of protein versus BCAAs from the senescence data set. (I, J) *n* = 6–8 mice/group. The overall effect of diet represents the *p*‐value from a two‐way ANOVA; *p < 0.05, Sidak's test post 2‐way ANOVA conducted separately for each gene. Data represented as mean ± SEM.

We randomized 12‐week‐old C57BL/6J male mice to these diets, following them longitudinally for 16 weeks (Figure 1A). As we anticipated based on our previous studies, we found that mice consuming diets low in BCAAs (i.e., LP, CP‐BR, HP‐BR) gained significantly less weight and fat mass than CP‐ and HP‐fed mice, with an overall reduction in adiposity (Figure S1A–H). Importantly, this weight reduction was not due to decreased calorie intake; mice consuming the LP, CP‐BR, and HP‐BR diets consumed more calories than CP and HP‐fed mice (Figure S1I–L). Due to this increase, animals consuming the CP‐BR and HP‐BR diets had an overall increase in calories derived from AAs, even while BCAAs were effectively restricted (Figure S1M–P).

We have previously observed that the paradoxical decreased weight gain of BCAA‐restricted mice is associated with increased energy expenditure (Cummings et al. 2018; Fontana et al. 2016). Using metabolic chambers, we determined energy expenditure via indirect calorimetry while also assessing food consumption, activity, and fuel source utilization. As we anticipated, mice with reduced levels of BCAAs had increased energy expenditure relative to the respective BCAA‐replete diet (Figure S2A–E). We also noted a tendency for increased spontaneous activity in animals with lower levels of BCAAs that reached statistical significance in some cases (Figure S2F).

Increased energy expenditure in LP‐fed mice is mediated by FGF21, which is induced by PR and stimulates the beiging of inguinal white adipose tissue (iWAT) and the activation of brown adipose tissue (BAT) by stimulating sympathetic nerve activity (Hill et al. 2017; Laeger et al. 2014; Owen et al. 2014). We observed that BCAA restriction increased plasma FGF21 in the context of the CP (21% protein) diet, but not the HP diet (36% protein diet) (Figure S2G,H). We observed that thermogenic genes were upregulated in the inguinal white adipose tissue (iWAT) in response to an LP diet, but not in response to BCAA restriction; conversely, in BAT, we observed a BCAA‐restriction induction of several thermogenic genes (Figure S2I,J). Mice consuming diets low in BCAAs have a higher respiratory exchange ratio (RER), suggesting an increase in carbohydrate/protein substrate utilization (Figure S2K–M). This data is consistent with BCAA restriction increasing energy expenditure via FGF21‐induced activation of thermogenesis.

We also assessed glucose homeostasis by performing glucose, insulin, and alanine tolerance tests. Mice consuming LP and low BCAA diets had improved glucose and pyruvate tolerance and lower fasting blood glucose relative to mice consuming CP and HP diets; however, insulin sensitivity was improved only in LP‐fed mice (Figure S3A–G).

### 3.2 LP‐ and CP‐BR‐Fed Animals Decreased Hepatic Cellular Senescence

As described above, we hypothesized that the metabolic benefits of low BCAA diets are in part through altered senescence. We therefore examined the mRNA expression of common senescence and SASP genes. We observed that BCAA restriction reduced the expression of the senescence marker *p16*, while the expression of *p21* was not significantly altered (Figure 1B,C). We also performed hepatic SA‐β‐Gal staining and livers from mice fed diets with low levels of BCAAs (LP, CP‐BR, and HP‐BR) had lower SA‐β‐Gal‐positive staining than livers from CP and HP‐fed mice (Figure 1D,E). BCAA restriction also had strong effects on the expression of multiple SASP genes, reducing the expression of *Il1α, Il6, Ccl2*, and *Cxcl1*, while not affecting the expression of several other SASP genes (Figure 1F,G; Figure S4A–D).

The effects of BCAA restriction were generally stronger in the context of the CP diet, while BCAA restriction in the context of the HP diet either had no effect or did not reach statistical significance. To better understand the roles of BCAAs and protein in the regulation of these senescence and SASP genes, we analyzed the mRNA expression using a Multiple Linear Regression (MLR) approach with gene expression as the dependent variable and the level of either dietary protein or BCAAs as the independent variables. We found that dietary BCAAs substantially contributed to the expression of most of the senescence and SASP genes we examined, while dietary protein level did not (Figure 1H).

We examined the effects of BCAA restriction on senescence and the SASP in other contexts to determine the general applicability of these findings. We found that there was an overall beneficial effect of BCAA restriction on senescence and SASP gene expression in the livers of aged C57BL/6J male mice, as shown by the significant overall effect of diet, with restriction of BCAAs significantly reducing expression of *p16* (Figure 1I). We further found that BCAA restriction had an overall beneficial effect on senescence and SASP gene expression in the livers of mice fed a high‐fat, high‐sucrose Western diet, reducing expression of *p16*, *p21*, and *Il1α* (Figure 1J).

### 3.3 Low BCAA Diets Display a Tissue‐Specific Effect on Adipose Tissue

We repeated this study in p16‐3MR mice. Overall, the effects of protein and BCAAs on body weight, food intake, and glucose regulation in p16‐3MR males were similar to what we observed in C57BL/6J males (Figure S5A–O).

**FIGURE 2:**
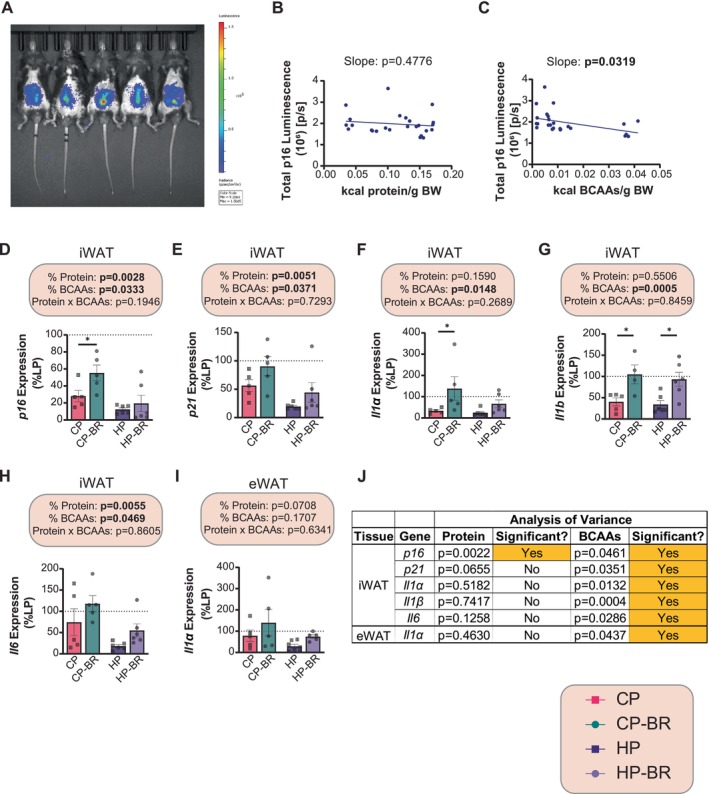
Diets low in BCAAs promote senescent cell accumulation in the adipose tissue of p16‐3MR male mice. (A) In vivo bioluminescence of p16 after 22 weeks on the indicated diets (A). (B, C) Linear regression of total p16 luminescence versus kcals of protein (B) and kcals of BCAAs (C) consumed. (D, H) iWAT mRNA expression of the indicated genes. (I) eWAT mRNA expression of *Il1α*. (J) Multiple Linear Regression (MLR) analysis of significant adipose tissue senescence genes. (B, C) Linear regression of total p16 luminescence as a function of kilocalories from either protein (B) or BCAAs (C); slope displayed to determine relationship. (D, I) *n* = 4–6 mice/group (100% = average expression of the indicated gene in the iWAT or eWAT of LP‐fed mice); the overall effect of protein, BCAAs, and the interaction represent the *p*‐value from a two‐way ANOVA; *p < 0.05, Sidak's test post 2‐way ANOVA. (J) Statistics for the *p*‐value are from a MLR analysis to determine the contribution of protein versus BCAAs from the senescence data set. Data represented as mean ± SEM.

At 20–22 weeks of age, we analyzed p16 expression in vivo using a bioluminescence assay. Surprisingly, we found no significant difference between mice fed any of the diets on whole‐body total luminescence, which represents p16 expression (Figure 2A and Figure S5P). We then plotted the total p16 luminescence against the calories of either protein or BCAAs consumed and performed a simple linear regression; a slope *p*‐value of less than 0.05 suggests that there is a linear relation between the variables. While there was no relationship between p16 luminescence and the calories of protein consumed, we found a statistically significant relationship between the calories of BCAAs consumed and p16 luminescence. Surprisingly though, this relationship was negative, with p16 luminescence decreasing as BCAA consumption increased (Figure 2B,C).

We therefore decided to examine the adipose depots, specifically, the iWAT and epididymal white adipose tissue (eWAT) since they are likely to show bioluminescence in the abdominal area, where we detected strong p16 activity, and diets have been shown to have a robust effect on adipose tissue senescence in other studies (Ogrodnik et al. 2021; Ogrodnik et al. 2019; Allyson K. Palmer et al. 2019; Wang et al. 2022). In contrast to our original hypothesis that BCAA restriction would reduce senescence, in our original C57BL/6J mice we found a strong increase in the expression of multiple senescence and SASP genes in BCAA‐restricted mice, with the expression of *p16, p21, Il1α, Il1β*, and *Il6* increasing in BCAA‐restricted iWAT, and *Il1α* increasing in BCAA‐restricted eWAT (Figure 2D–I). This interpretation was further supported by MLR analysis, which found a strong contribution of BCAA levels—but not protein level—to the expression of these senescence and SASP genes (Figure 2J). Broadly, we find that opposed to what we observed in the liver, BCAA restriction increases senescence in adipose tissue, increasing expression of most senescence and SASP genes in iWAT, increasing expression of *Il1α* expression in eWAT, and increasing some senescence genes in BAT as well (Tables S3 and S4).

The differential effect of BCAAs on senescence in the liver and iWAT was quite surprising. We hypothesized that this might be due in part to the differential effect of FGF21, a hormone induced by protein restriction and BCAA restriction that has been shown to regulate senescence (Li et al. 2019; Lu et al. 2021). FGF21 has differential effects on mTOR protein kinase signaling in liver and adipose tissue (Minard et al. 2016), and mTOR signaling is a key regulator of aging, metabolism, and components of the senescence and SASP (Calubag et al. 2024). We examined *Fgf21* expression in the liver and three adipose depots; *Fgf21* expression was higher in the liver and iWAT of LP‐fed mice than in all other groups, with CP‐BR‐fed mice having the next highest expression; there were minimal effects of BCAAs on *Fgf21* expression in eWAT or BAT (Figure S7A–D). Therefore, we then looked at mTORC1 signaling in the liver and iWAT. There were minimal effects of diet on the phosphorylation of the mTORC1 substrates S6K1 T389 or 4E‐BP1 T37/S46, or of the downstream readout S6 S240/S244 in either of the tissues (Figure S7E–J).

**FIGURE 3:**
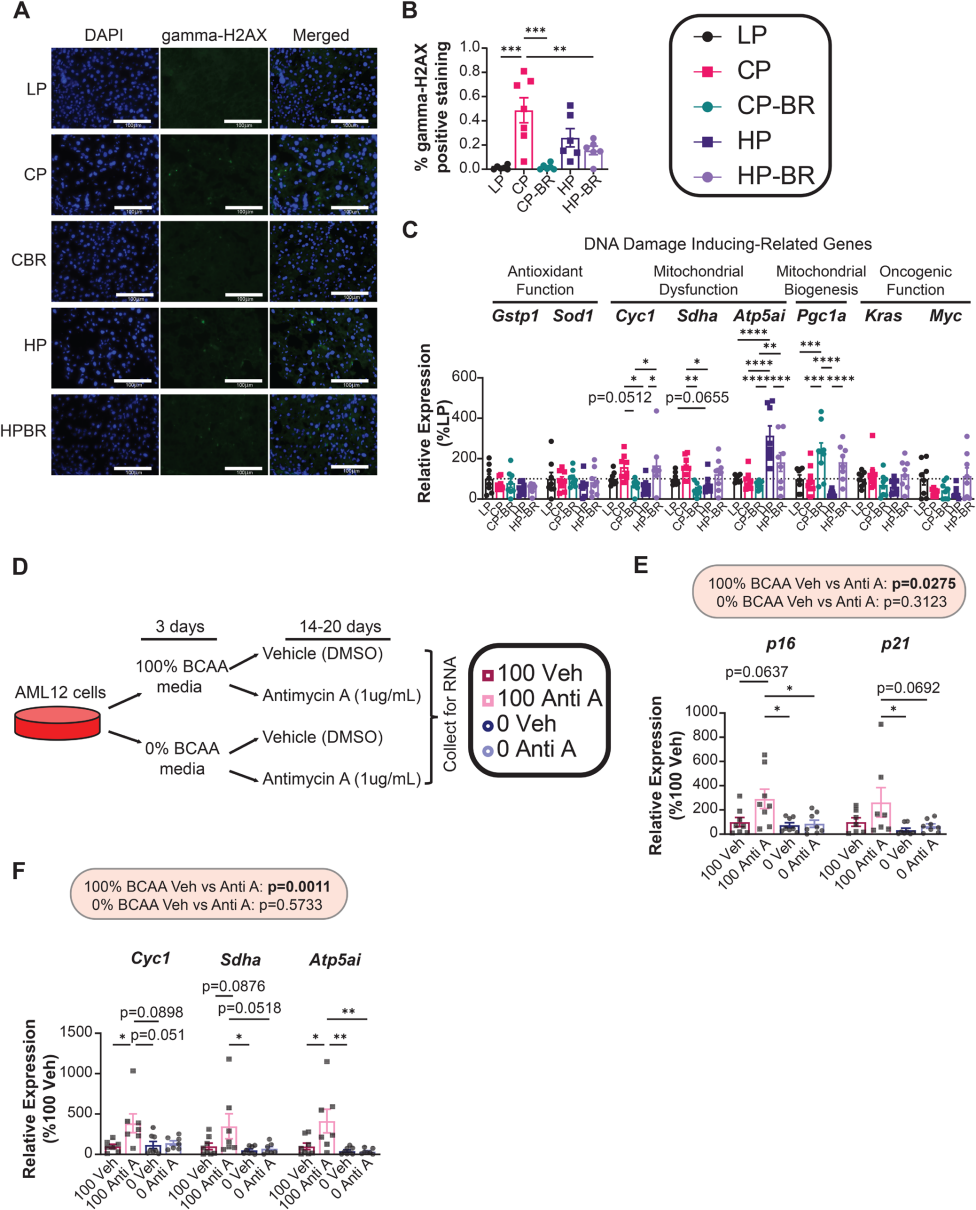
Lowering BCAAs protect from mitochondrial‐dysfunction‐related senescence in hepatocytes. (A, B) Representative images of hepatic gamma‐H2AX immunofluorescence staining (A) and quantification of gamma‐H2AX‐positive area (B). *n* = 6–7 mice/group; *p < 0.05, Tukey test post ANOVA. (C) Hepatic mRNA expression of DNA‐damage‐related genes. *n* = 5–8 mice/group; *p < 0.05, Tukey test post 2‐way ANOVA conducted separately for each gene. (D) Experimental design for AML12 cell culture. (E‐F) mRNA expression of *p16* and *p21* (E) and mitochondrial‐related genes (*Cyc1, Sdha* and *Atp5a1*) (F) in AML12 cell culture. (E, F) *n* = 8 biological replicates/group; the overall effect of Antimycin A (Anti A) and BCAA level in media from a 2‐way ANOVA conducted separately for 100% BCAAs and 0% BCAAs; *p < 0.05, **p < 0.01, ***p < 0.001, ****p < 0.0001, Tukey test post 2‐way ANOVA conducted separately for each gene. Data represented as mean ± SEM.

To gain insight into what types of damage might be signaling liver and iWAT cells to undergo senescence, we looked at staining and several genes related to DNA damage. First, we confirmed that there was increased DNA damage using gamma‐H2AX immunofluorescence staining in the liver of the CP‐fed mice compared to the low BCAA diets (Figure 3A,B). We then found that a BCAA‐restricted diet affected the expression of genes related to mitochondrial dysfunction and mitochondrial biogenesis, without altering the expression of genes related to antioxidant function and oncogenesis in both the liver and adipose tissue (Figure 3C; Figure S6A). Specifically, we found that the livers of CP‐BR‐fed mice had lower *Cycl1*, *Sdha*, and *Atp5ai* expression as well as increased *Pgc1a* expression compared to CP‐fed mice, suggesting they had improved mitochondrial function (Figure 3C). In contrast, we found the opposite gene expression changes in *Cyc1* and *Sdha* in the iWAT of CP‐BR‐fed mice, suggesting they had impaired mitochondrial function (Figure S6A).

This suggested to us that BCAAs, specifically at 21% protein, may mediate mitochondrial function/dysfunction, leading to the tissue‐specific differences in senescence. To confirm that mitochondrial dysfunction related to dietary BCAA may be mediating the effects on senescence, we turned to cell culture. We cultured mouse AML12 hepatocytes in media containing the normal level of BCAAs (100% BCAA) or restricted BCAAs (0% additive BCAA; this media still contains some BCAAs from serum). We treated these cells with antimycin A for 14–20 days to induce mitochondrial dysfunction‐related senescence, and then collected the cells to assess senescence (Figure 3D). As we expected, AML12 cells treated with antimycin A senesced, with increased expression of *p16* and *p21* (Figure 3E). Culture of cells in the BCAA‐restricted media (0% added BCAA) prevented antimycin βA‐induced senescence. This difference in senescence was associated with antimycin A‐induced mitochondrial dysfunction, which occurred in antimycin A‐treated cells cultured in 100% BCAA, and cells cultured in 0% BCAA media were protected from (Figure 3F). These results support a model in which BCAA restriction protects liver cells from senescence via cell‐autonomous effects on mitochondrial dysfunction‐related senescence induction.

To further explore the tissue‐specific effects of BCAAs on senescence, we explored the possibility of lipolysis playing a role. It was recently shown that DNA damage leads to altered mitochondrial structure and dynamics, which downregulates fatty acid oxidation (the first step of which is lipolysis) and induces cellular senescence (Yamauchi et al. 2024). In contrast, the induction of systemic lipolysis in *Drosophila* has been shown to reduce senescence in the ovary and gut (Shang et al. 2022). Therefore, we looked at gene expression of lipolysis in both the liver and iWAT and found that at least at 36% protein, lipolytic gene expression was increased in the liver and decreased in the iWAT by BCAA restriction (Figure S7K,L). This suggests that the increased lipolysis in the liver and reduced lipolysis in the iWAT in response to BCAA restriction may also be playing a role in the induction of senescence, particularly in the 36% protein context.

### 3.4 Restriction of Each Individual BCAA Does Not Replicate the Effects of Restricting All Three BCAAs on Hepatic Cellular Senescence

The metabolic benefits of the CP‐BR diet are due primarily to the restriction of isoleucine, with a lesser contribution from the restriction of valine (Yu et al. 2021). To determine if the effects of CP‐BR on cellular senescence are due to a single BCAA, we placed 12‐week‐old C57BL/6J male mice on the CP and CP‐BR diets, as well as on leucine‐restricted (Leu‐R), isoleucine‐restricted (Ile‐R) or valine‐restricted (Val‐R) diets. All of these diets are isocaloric with identical levels and sources of fats and carbohydrates and are isonitrogenous through the addition of non‐essential amino acids in restricted diets; the full diet composition is provided in Table S1.

**FIGURE 4:**
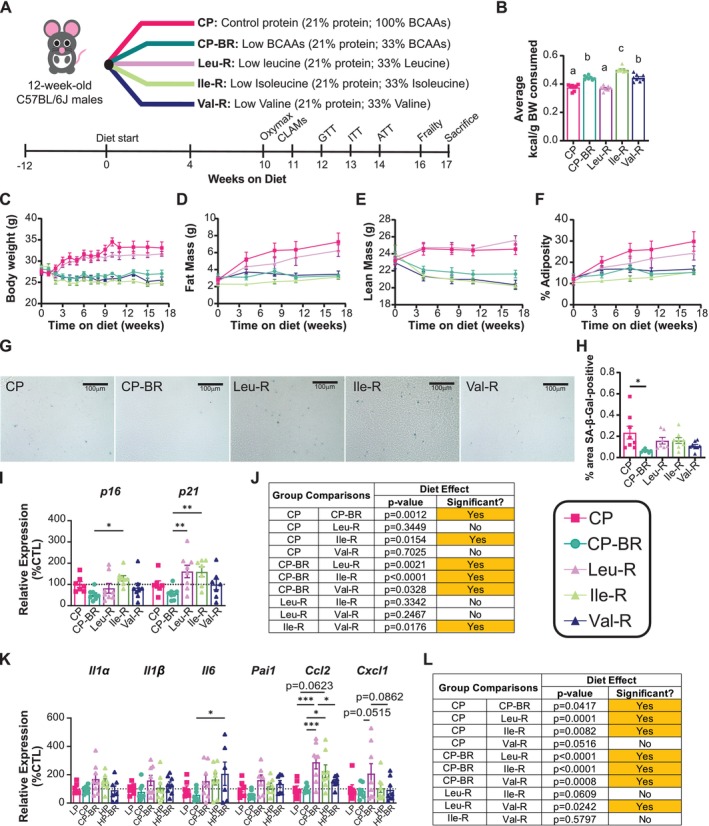
Restriction of each individual BCAA does not replicate the effects of a CP‐BR diet on hepatic cellular senescence. (A) Experimental design. (B) Average consumption of kilocalories per gram of body weight (kcal/g BW) per group over 17‐week period on diet. (C, F) Body weight (C), fat mass (D), lean mass (E), and adiposity (F) of mice fed the indicated diets over time. (G, H) Hepatic SA‐β‐Gal staining at 40X magnification (scale bar = 100 μm) (G) with quantification of SA‐β‐Gal‐positive area (H). (I, J) Hepatic mRNA expression of *p16* and *p21* (I) and the overall effect of each diet on senescence gene expression (J). (K, L) Hepatic SASP mRNA expression (K) and the overall effect of each diet on SASP gene expression (L). (B) *n* = 7–8 mice/group; means with the same lowercase letter are not significantly different from each other, Tukey test post ANOVA, *p* < 0.05. (H) *n* = 7–8 mice/group; *p < 0.05, Tukey test post ANOVA. (I, L) *n* = 8 mice/group; the overall effect of diet from a 2‐way ANOVA; *p < 0.05, **p < 0.01, ***p < 0.001, Tukey's test post 2‐way ANOVA conducted separately for each gene. Data represented as mean ± SEM.

Over the course of 17 weeks, we tracked physiological parameters in all groups of mice (Figure 4A). As anticipated, CP‐BR‐, Ile‐R‐, and Val‐R‐fed mice consumed more food than CP and Leu‐R‐fed mice, yet these groups showed attenuated weight and fat mass gain (Figure 4B–F and Figure S8A–D) as a result of increased energy expenditure (Figure S8F–H). These phenotypes were associated with strong induction of FGF21 in the blood of CP‐BR, Ile‐R, and Val‐R‐fed mice and increased expression of the thermogenic genes *Bmp8b* and *Elovl3* in the BAT of CP‐BR‐ and Val‐R‐fed mice (Figure S8I,J), and with improved blood glucose control in the same animals as well as Ile‐R‐fed mice (Figures S8L–R).

Finally, we assessed hepatic senescence. Although CP‐BR did reduce SA‐β‐Gal positivity and senescence gene expression, Ile‐R and Val‐R did not, despite their improvements in metabolic health (Figure 4G–L). Interestingly, restriction of individual BCAAs seemed to increase, not decrease, hepatic SASP expression (Figure 4K,L). This suggests that while Ile‐R and Val‐R may mediate the metabolic and physiological effects seen in CP‐BR diets, they do not mediate the effects of BCAA restriction on hepatic senescence and the SASP at this age.

### 3.5 The Effect of BCAAs on Metabolism and Cellular Senescence Is Sex‐Specific

**FIGURE 5:**
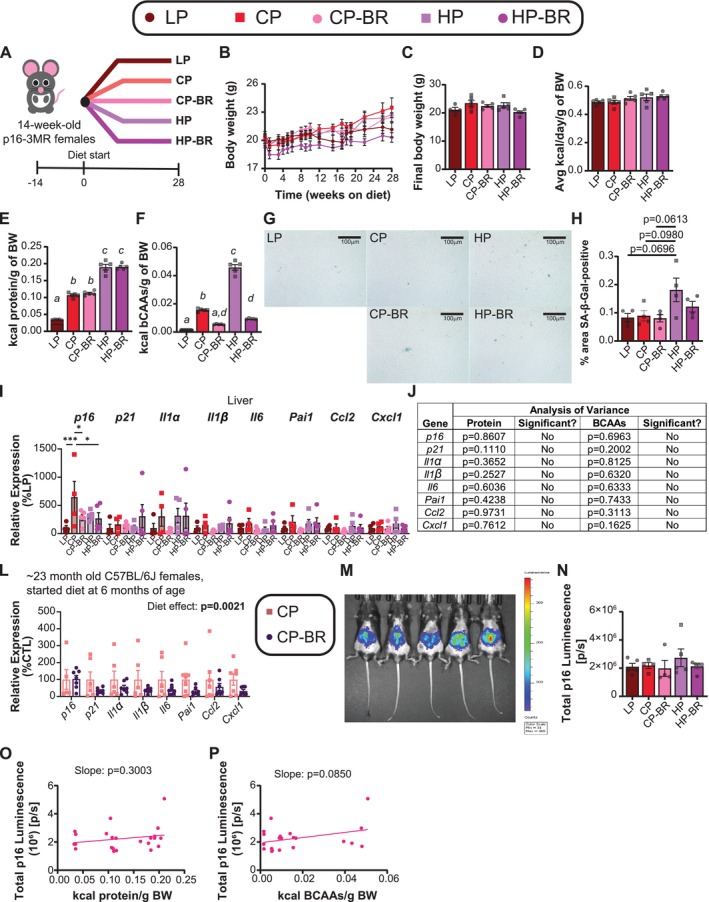
Low BCAA diets do not have the same benefits in female mice as they do in male mice. (A) Experimental design. (B, C) Body weight of mice fed the indicated diets over time (B) and final body weights (C). (D) Average kilocalories consumed per gram of body weight by each group of mice over the entire study. (E, F) Average kilocalories derived from protein (E) or derived from BCAAs (F) consumed by each group of mice over the entire study. (G, H) Hepatic SA‐β‐Gal staining at 40X magnification (scale bar = 100 μm) (G) with quantification of SA‐β‐Gal‐positive area (H). (I, J) Hepatic mRNA expression of senescence and SASP genes (I) and Multiple Linear Regression (MLR) analysis to determine contribution of protein versus BCAAs to gene expression (J). (L) Hepatic mRNA expression of senescence and SASP genes in female C57BL6/J mice fed the CP and CP‐BR diets for ~7 months (from 16 to 23 months of age). (M–N) In vivo bioluminescence of p16 after 22 weeks on the indicated diets (M); total p16 luminescence was quantified (N). (O, P) Linear regression of total p16 luminescence versus kcals consumed of either protein (O) or BCAAs (P). (B, J, M, P) *n* = 4–5 female mice/group. (L) *n* = 5–7 female mice/group. (C, F, H, N) means with the same lowercase letter are not significantly different from each other, Tukey test post ANOVA, *p* < 0.05. (I) *p < 0.05, Tukey test post 2‐way ANOVA conducted separately for each gene. (J) Statistics for the *p*‐value are from a MLR analysis to determine the contribution of protein versus BCAAs from the senescence data set. (I) *p < 0.05, Sidak's test post 2‐way ANOVA conducted separately for each gene. (O, P) Linear regression of total p16 luminescence as a function of kilocalories from either protein (O) or BCAAs (P); slope displayed to determine relationship. Data represented as mean ± SEM.

To examine the role of sex in the response of senescence to dietary protein, we placed 14‐week‐old, female, p16‐3MR mice on the five diets (LP, CP, CP‐BR, HP, HP‐BR) for 28 weeks (Figure 5A). After 28 weeks on diet, we found largely no effect of diet in females (Figure 5B,C). In agreement with this, BCAA‐restricted females do not consume more food, but their consumption of protein and BCAAs was similar to that of the males (Figure 5D–F; Figures S9A–C and S1M–P). While females fed a low BCAA diet tended to have improved glucose tolerance (Figure S9D,E), BCAAs largely had no effect on fasting blood glucose, insulin sensitivity, nor suppression of hepatic gluconeogenesis (Figure S9F,J).

We next examined senescence in the livers of female p16‐3MR mice. We found no significant difference in SA‐β‐Gal activity between any of the groups, though HP‐fed females trended toward higher hepatic SA‐β‐Gal activity than LP‐, CP‐, and CP‐BR‐fed mice (*p* = 0.0696, *p* = 0.0980, and *p* = 0.0613, respectively) (Figure 5G,H), suggesting that higher protein consumption may induce senescence in females. While LP, CP‐BR, and HP‐BR‐fed females did have reduced expression of *p16* compared to CP‐fed females, this was the only significant difference identified; using the MLR approach we used previously, we found that neither dietary BCAAs nor dietary protein contributed to the expression of any of the senescence and SASP genes we examined (Figure 5I,J). While females are thought to be more prone to senescence (Ng and Hazrati 2022; Yousefzadeh et al. 2020), it may not be the case in young animals, such as in this study, as we see higher SA‐β‐Gal‐positive staining in the liver of the males compared to females (Figures 1D,E and 5G,H). It may also be that in young mice, female‐specific senescence accumulation is tissue‐specific, and we would have seen senescence in other tissues (Jin et al. 2023). As LP and CP‐BR diets reduced *p16* expression in the liver and we saw trends in reduced SA‐β‐Gal‐positive staining (Figure 5H,I), we decided to look at CS senescence and SASP gene expression in the livers of C57BL/6J female mice fed a CP‐BR diet for ~7 months (from 16 months of age until they were 23 months of age), using mRNA banked from a previous study (Richardson et al. 2021). We found that there was an overall significant effect of diet on senescence and SASP gene expression (Figure 5L), suggesting that a reduction of dietary BCAAs can reduce hepatic senescence in middle‐aged/aged C57BL/6J females.

We also assessed p16 bioluminescence; we observed no significant differences in p16 expression as quantified by total p16 luminescence in the females (Figure 5M,N). While there was a trending positive relationship between luminescence and BCAAs consumed (*p* = 0.085), we did not see significant effects of BCAAs when performing a MLR analysis in the iWAT (Figure 5O,P and Figure S9K,L).

Overall, we found a sex‐specific effect of BCAAs on metabolic health and senescence, with female mice largely not benefiting from BCAA restriction with respect to the effects on weight, body composition, and hepatic cellular senescence.

## 4 Discussion

Senescence is one of the hallmarks of aging (Lopez‐Otin et al. 2013), and the long‐term accumulation of senescent cells has negative consequences on health and aging (Calubag et al. 2024). Dietary interventions can protect against senescence, with some studies showing that specific dietary macronutrients can impact senescence (Calubag et al. 2024; Nehme et al. 2021; Ogrodnik et al. 2017, 2019; Wang et al. 2022). Dietary protein can promote hepatic senescence (Nehme et al. 2021), and recent studies have shown that impaired catabolism of the BCAAs promotes senescence (Han et al. 2023; Liang et al. 2023).

Here, we have examined how dietary BCAAs and protein impact both metabolic health and CS. We find that diets low in BCAAs (LP, CP‐BR and HP‐BR) normalize body weight, preventing body weight gain and adiposity accretion, regardless of the overall level of dietary protein, in male mice. This effect is mediated by changes in energy balance, with restriction of BCAAs boosting food intake as well as energy expenditure via increased thermogenesis in the iWAT of LP‐fed mice and in the BAT of CP‐BR‐fed and HP‐BR‐fed male mice. Male mice consuming diets with reduced levels of BCAAs also have improved glucose tolerance. Female mice generally have a substantially blunted response to reduction of BCAAs.

We see strong effects of BCAA restriction on senescence in the liver, with a reduction in DNA damage and SA‐β‐Gal staining as well as senescence and SASP gene expression, with a robust effect in males under a variety of diets and ages as well as in aged females. Notably, our findings show that dietary protein content itself is not otherwise associated with hepatic senescence or SASP gene expression, except insofar as higher protein diets normally contain higher levels of BCAAs. However, restricting BCAAs in the context of a HP diet does not protect against senescence to the same extent as it does in the CP diet, suggesting that other components of the overall dietary context—for example, the decreased levels of carbohydrates in the HP diet—may impact the effect of BCAAs on hepatic senescence. As the metabolic benefits of lower BCAAs are not lower in HP‐fed mice, the benefits of BCAA restriction for senescence are uncoupled from its effects on metabolic health.

The benefits of lower levels of BCAAs on senescence in the liver are cell‐autonomous, with BCAA restriction protecting the AML12 mouse hepatocyte cell line from mitochondrial dysfunction‐induced senescence. However, the liver is a heterogeneous organ with many different cell types, and we did not assess the effects of BCAA restriction on senescence in other cell types, nor did we assess signaling between cell types. Kupffer cells, which are anti‐inflammatory M2 macrophages, have been shown to promote senescence in the liver to protect from alcoholic liver disease (Wan et al. 2014). Senescent immune cells have also been shown to promote senescence in other solid organs (Yousefzadeh et al. 2021). Additionally, senescent liver sinusoid endothelial cells (LSECs) have been shown to contribute to liver steatosis and metabolic dysfunction (Duan et al. 2023; Grosse and Bulavin 2020). Future work could also explore if Kupffer, LSEC, or other cell populations in the liver may also contribute to the effects of BCAAs on cellular senescence in the liver.

In contrast to hepatic senescence, we found that lowering dietary BCAAs promotes senescence in iWAT. It may be that there is some protective impact of senescence in fat, at least in lean mice as studied here. Senescence is important for wound healing (Kita et al. 2022), and perhaps the fat depot is in a state of healing. Similar to the liver, the adipose tissue is comprised of many cell types, and the stromal vascular fraction, which is made up of many cell types including pre‐adipocytes, immune cells, and endothelial cells, contains more senescent cells than the adipocytes themselves (Wang et al. 2022). Thus, it is possible that the changes in iWAT senescence we observed result not from changes in adipocytes, but from other cell types such as immune cells or endothelial cells (Feng et al. 2023; Shimi et al. 2024). Further research is needed to assess which cell types are becoming senescent in the iWAT.

The mechanisms by which BCAA restriction promotes senescence in the iWAT is also unclear. One possibility is that lipolysis in the liver leads to the release of free fatty acids and accumulation in the adipose tissue, leading to dysfunctional iWAT and increased senescence. Another possibility is that a reduction in lipolysis in the iWAT promotes metabolic dysregulation and senescence (Xiang et al. 2020). Interestingly, a link between senescence in the liver and adipose tissue has recently been reported by another group, which found that the clearance of senescent cells in the adipose tissue results in decreased senescence in the liver (Tang et al. 2024). Additional research will be required to fully understand how lower levels of BCAAs protect against hepatic senescence, the relationship between the liver and iWAT in response to BCAA‐mediated senescence, and why lower BCAAs potentiate senescence in the iWAT without impairing metabolic health. It will also be informative to identify the effects of dietary BCAAs on senescence in other tissues and cell types, and understand how sex interacts with BCAAs to influence CS.

Limitations of our study include that these studies were primarily conducted in young, male mice. We chose to conduct the study in this way based on the results of a previous study of protein consumption and senescence (Nehme et al. 2021), but more pronounced differences may have been observed if we had used older mice with a higher spontaneous rate of senescence or placed the mice on these diets for a longer period of time. Our mice were primarily sacrificed in the refed state following an overnight fast, and assessing tissues in a different feeding state could also provide additional insights. These studies were conducted exclusively in C57BL/6J mice, and we have shown that strain as well as sex contribute to the metabolic response to PR (Green, Pak, et al. 2022). Future studies should test the robustness of our results in other mouse strains as well as in genetically heterogeneous mice. We also did not examine the plasma levels of BCAAs in this study, but in our previous studies, we have found that dietary restriction of either all three BCAAs or the individual BCAAs does not reduce plasma levels of BCAAs (Yu et al. 2021). This agrees with previous work by our lab and others showing that blood levels of AAs are defended against protein restriction in both mice and humans (Fontana et al. 2016; Yap et al. 2020). Lastly, we also limited our cell culture experiments to AML12 cells. As there are many other cell types within the liver, many of the effects on hepatic senescence we observed may have been due to the effects of BCAAs on other cell types.

The fact that we did not see as strong of an effect in females is quite striking, yet unsurprising, because this mimics the other male‐specific lifespan extension benefits seen in other dietary restrictions of protein and amino acids (Green et al. 2023; Hill et al. 2022; Richardson et al. 2021). Studies of calorie restriction, protein restriction, and geroprotective drugs like rapamycin have shown that different levels of restriction or drugs can yield different responses (Green, Pak, et al. 2022; Miller et al. 2014; Mitchell et al. 2016; Solon‐Biet et al. 2014; Weindruch et al. 1986). Perhaps the level of restriction and protein we chose to conduct our study is not optimal for females, and future studies should test how different levels of restriction impact females.

Additionally, we conducted our female studies and some of our male studies in p16‐3MR mice. A recent report suggests that contrary to earlier reports, p16‐3MR mice do not show significant bioluminescent changes with aging, doxorubicin treatment, or during wound healing (Hori et al. 2024). While we did see some trends in bioluminescence, particularly when we plotted flux against BCAA consumption, we acknowledge that there may be flaws in this mouse model.

In summary, recent work from multiple labs has shown that calorie quality, not just the total quantity, is a critical determinant of biological health, and that dietary protein in particular is a critical mediator of healthy aging (Green, Lamming, et al. 2022; Mihaylova et al. 2023). We and others have shown that many beneficial effects of low protein diets are mediated by reduced levels of the BCAAs, and that dietary levels of BCAAs are negatively associated with lifespan (Richardson et al. 2021; Solon‐Biet et al. 2019). Here, we find that reducing dietary BCAAs reduces hepatic senescence via a cell‐autonomous effect that is uncoupled from the beneficial effects of BCAA restriction on metabolic health. Surprisingly, the effects of BCAA restriction are tissue‐specific, and while low dietary BCAAs reduce hepatic senescence, they increase senescence in adipose tissue. Overall, these data support the notion that dietary composition is a critical regulator of senescence burden during aging, and highlight BCAAs as the critical mediator of the effects of dietary protein on senescence.

## Author Contributions

Experiments were performed in the Lamming laboratory. M.F.C., A.R.K., D.A.H., and D.W.L. conceived the experiments and secured funding. M.F.C., I.A., I.D.G., S.M.L., P.L., L.E.B., R.B., M.M.S., J.A.I., B.A.K., and F.X. performed the experiments. M.F.C., I.A., P.L., L.E.B., S.M.L., D.M., and D.W.L. analyzed the data. M.F.C., A.R.K., D.A.H., and D.W.L. wrote and edited the manuscript.

## Conflicts of Interest

D.W.L. has received funding from, and is a scientific advisory board member of, Aeovian Pharmaceuticals, which seeks to develop novel, selective mTOR inhibitors for the treatment of various diseases.

## Supporting information


**Data S1.** Raw values for all graphs.

**Data S2.** Source Images for Senescence‐Associated β‐Galactosidase Staining and Western blots.

**Data S3.** Source Images for gamma‐H2AX staining in Figure 3A.

**Figure S1.** Diets low in BCAAs normalize body weight without malnutrition. (A, B) Body weight of mice fed the indicated diets over time (A) and final body weight at end of study (B). (C, D) Fat mass over time (C) and final fat mass (D). (E, F) Lean mass over time (E) and final lean mass (F). (G, H) Percent adiposity over time (G) and final percent adiposity (H). (I, J) Kilocalories consumed over time (I) and average kilocalories consumed over the course of the entire study (J). (K, L) Kilocalories consumed per gram of body weight over time (K) and average kilocalories per gram of body weight consumed over the course of the entire study (L). (M, N) Kilocalories per gram of body weight derived from protein over time (M) and average kilocalories per gram of body weight consumed over the course of the entire study (N). (O, P) Kilocalories per gram of body weight derived from BCAAs over time (O) and average kilocalories per gram of body weight consumed over the course of the entire study (P). (A, P) *n* = 10 mice/group. (B, D, F, H, J, L, N, P) means with the same lowercase letter are not significantly different from each other, Tukey test post ANOVA, *p* < 0.05. Data represented as mean ± SEM.
**Figure S2.** Restriction of BCAAs increases energy expenditure via brown adipose tissue thermogenesis. (A) Average light and dark energy expenditure. (B, E) Average energy expenditure over 24‐h period as a function of body weight (B, C) or lean mass (D, E). (F) Total spontaneous activity over 24‐h period per group. (G, H) Circulating FGF21 in the fasted (G) and refed (H) state. (I, J) mRNA expression of thermogenic genes in iWAT (I) and BAT (J). (K, L) Respiratory exchange ratio (RER) over 24‐h period represented in Zeitgeber hours (K) and their averaged values (L). (M) Fuel oxidation of fats and carbohydrates or protein. (A, H, K, M) *n* = 8–10 mice/group; collected during Weeks 8–9. (A) means with the same lowercase letter are not significantly different from each other, Tukey test post ANOVA conducted separately for the light and dark cycles, *p* < 0.05. (C, E) simple linear regression (analysis of covariance) was calculated to determine if the slopes or elevations are equal; if the slopes are significantly different, differences in elevation cannot be determined. (F) *p < 0.05, Tukey test post ANOVA. (G, H) means with the same lowercase letter are not significantly different from each other, Tukey test post ANOVA, *p* < 0.05. (I) *n* = 5–6 mice/group; means with the same lowercase letter are not significantly different from each other, Tukey test post 2‐way ANOVA conducted separately for each gene, *p* < 0.05. (J) *n* = 7–8 mice/group; *p < 0.05, Tukey test post 2‐way ANOVA conducted separately for each gene. (L, M) *p < 0.05, Tukey test post ANOVA conducted separately for the light and dark cycles (L) and for FAO/CPO (M). Data represented as mean ± SEM.
**Figure S3.** Diets low in BCAAs improve glucose homeostasis. (A, B) Glucose tolerance test conducted after 10 weeks on diet (A) and quantified area under the curve (B). (C, D) Suppression of hepatic gluconeogenesis as assessed by alanine tolerance test conducted after 12 weeks on diet (C) and quantified area under the curve (D). (E, F) Insulin tolerance test conducted after 11 weeks on diet (E) and quantified area under the curve (F). (G) 4, h fasting blood glucose levels were collected after 11 weeks on diet. (A‐G) *n* = 10 mice/group. (B, D) means with the same lowercase letter are not significantly different from each other, Tukey test post ANOVA, *p* < 0.05. (F, G) *p < 0.05, Tukey test post ANOVA. Data represented as mean ± SEM.
**Figure S4.** Hepatic SASP mRNA expression of male mice. (A, D) Hepatic mRNA expression of *Pai1*, *Il1β*, *Ccl2*, and *Cxcl1*. *n* = 8 mice/group. 100% = average expression of the indicated gene in the liver of LP‐fed mice. The overall effect of protein, BCAAs, and the interaction represent the *p*‐value from a two‐way ANOVA; *p < 0.05, Sidak’s test post 2‐way ANOVA. Data represented as mean ± SEM.
**Figure S5.** Diets low in BCAAs have similar benefits on metabolic health in p16‐3MR male mice. (A, B) Body weight over 28‐week period per group (A) and final body weight (B). (C, D) Kilocalories per gram of body weight consumed over time (C) and averaged over entire experiment (D). (E, F) Kilocalories derived from protein over time (E) and averaged (F). (G, H) Kilocalories derived from BCAAs consumed over entire study (G) and averaged (H). (I, J) Glucose tolerance test conducted after 12 weeks on diet (I) and quantified area under the curve (J). (K, L) Insulin tolerance test conducted after 13 weeks on diet (K) and quantified area under the curve (L). (M, O) Alanine tolerance test conducted after 14 weeks on diet (M) and quantified area under the curve (O). (P) Total in vivo p16 bioluminescence at 22 weeks on diet. (A‐P) *n* = 4–5 male mice/group. (B, J, L, O, P) *p < 0.05, Tukey test post ANOVA. (D, F, H) means with the same lowercase letter are not significantly different from each other, *p* < 0.05, Tukey test post ANOVA. (B, D, F, H, J, L, O, P) Data for each individual mouse is plotted; statistics for the *p*‐value from a one‐way ANOVA. Data represented as mean ± SEM.
**Figure S6.** Low BCAA diets increase iWAT expression of mitochondrial dysfunction‐related genes. (A) iWAT mRNA expression of DNA‐damage‐related genes. *n* = 5–6 mice/group; *p < 0.05, Tukey test post 2‐way ANOVA conducted separately for each gene.
**Figure S7.** Low BCAA diets do not induce a tissue‐specific effect on the FGF21/mTORC1 signaling pathway. (A, D) mRNA expression of Fgf21 in liver (A), iWAT (B), eWAT (C) and BAT (D). (E, J) Phosphorylation of S6 S240/S244 (E, H), S6K1 T389 (F, I) and 4E‐BP1 T37/S46 (G, J) in liver (E, G) and iWAT (H, J) via western blot. (K, L) mRNA expression of lipolysis genes in liver (K) and iWAT (L). (A, J) *n* = 4–8 mice/group. The overall effect of protein, BCAAs, and the interaction represent the *p*‐value from a two‐way ANOVA for the CP, CP‐BR, HP and HP‐BR groups only. means with the same lowercase letter are not significantly different from each other, Tukey test post 1‐way ANOVA, *p* < 0.05. (K, L) *n* = 4–6 mice/group; the overall effect of dietary BCAAs in the context of control protein (21%) and high protein (36%) from a 2‐way ANOVA conducted separately for 100% BCAAs and 0% BCAAs; *p < 0.05, Tukey test post 2‐way ANOVA conducted separately for each gene Data represented as mean ± SEM.
**Figure S8.** The benefits of CP‐BR on the metabolic health of C57BL/6J males are mediated by the restriction of isoleucine and valine. (A, D) Final body weight (A), fat mass (B), lean mass (C), and percent adiposity (D) after 17 weeks on diet. (E) Kilocalories per day per gram of body weight. (F) Energy expenditure per gram of body weight in the light and dark phase. (G‐H) Averaged energy expenditure over 24‐h period as a function of lean mass. (I) Circulating FGF21 in the fasted and refed state. (J) mRNA expression of thermogenic genes in BAT. (K) Total spontaneous activity over 24‐h period. (L, M) Glucose tolerance test conducted after 12 weeks on diet (L) and quantified area under the curve (M). (N, O) Insulin tolerance test conducted after 13 weeks on diet (N) and quantified area under the curve (O). (P) 4‐h fasting blood glucose levels taken after 13 weeks on diet. (Q, R) Suppression of hepatic gluconeogenesis as assessed by alanine tolerance test conducted after 14 weeks on diet (Q) and quantified area under the curve (R). (A, R) *n* = 8 mice/group. (A, D, M, R) means with the same lowercase letter are not significantly different from each other, Tukey test post ANOVA, *p* < 0.05. (F, I) means with the same lowercase letter are not significantly different from each other, Tukey test post ANOVA conducted separately for the light and dark cycles, *p* < 0.05. (H) simple linear regression (analysis of covariance) was calculated to determine if the slopes or elevations are equal; if the slopes are significantly different, differences in elevation cannot be determined. (J) *p < 0.05, Tukey test post 2‐way ANOVA conducted separately for each gene. (K, O, P) *p < 0.05, Tukey test post ANOVA, Data represented as mean ± SEM.
**Figure S9.** Diets low in BCAAs largely do not impact metabolic health in female mice. (A) Kilocalories per gram of body weight consumed over time. (B, C) Kilocalories consumed derived from protein (B) and BCAAs (C) over time. (D, E) Glucose tolerance test conducted after 12 weeks on diet (D) and quantified area under the curve (E). (F, G) Suppression of hepatic gluconeogenesis as assessed by alanine tolerance test conducted after 14 weeks on diet (F) and quantified area under the curve (G). (H, I) Insulin tolerance test conducted after 13 weeks on diet (H) and quantified area under the curve (I). (J) 4‐h fasting blood glucose levels taken after 13 weeks on diet. (K, L) mRNA expression of senescence genes in iWAT (K) with the statistics of contribution of protein and BCAAs to their expression (L). (A, L) *n* = 4–5 female mice/group. (E, G, I, J) means with the same lowercase letter are not significantly different from each other, Tukey test post ANOVA, *p* < 0.05. (K) *p < 0.05, Tukey test post 2‐way ANOVA conducted separately for each gene. (L) Statistics for the *p*‐value are from a Multiple Linear Regression (MLR) analysis to determine the contribution of protein versus BCAAs from the senescence data set. Data represented as mean ± SEM.

**Table S1.** Experimental Diets. The composition and calorie content of the experimental diets used in this study.
**Table S2.** qRT‐PCR primer sequences. Forward and reverse primer sequences for qRT‐PCR.
**Table S3.** Interaction, gene effect and diet effect in the liver, iWAT, eWAT, and BAT on senescence gene expression. Values between indicated groups represent the *p*‐value from a two‐way ANOVA; *p < 0.05, Sidak’s test post 2‐way ANOVA. *n* = 5–8 mice/group.
**Table S4.** Multiple linear regression analysis values from iWAT, eWAT and BAT. Multiple Linear Regression (MLR) analysis *p*‐value for the contribution of protein versus BCAAs in three adipose depots. *n* = 5–8 mice/group.
**Table S5.** Cell culture media composition. Cell culture media components and their catalog numbers.

## Data Availability

The data that supports the findings of this study are available in the supporting information—S1 of this article.
