# Supplementary material for: Tissue‐Specific Effects of Dietary Protein on Cellular Senescence Are Mediated by Branched‐Chain Amino Acids
Source: Aging Cell. 2025 Jul 28;24(10):e70176. doi: 10.1111/acel.70176 (PMC12507398; doi:10.1111/acel.70176)

Figure 1D

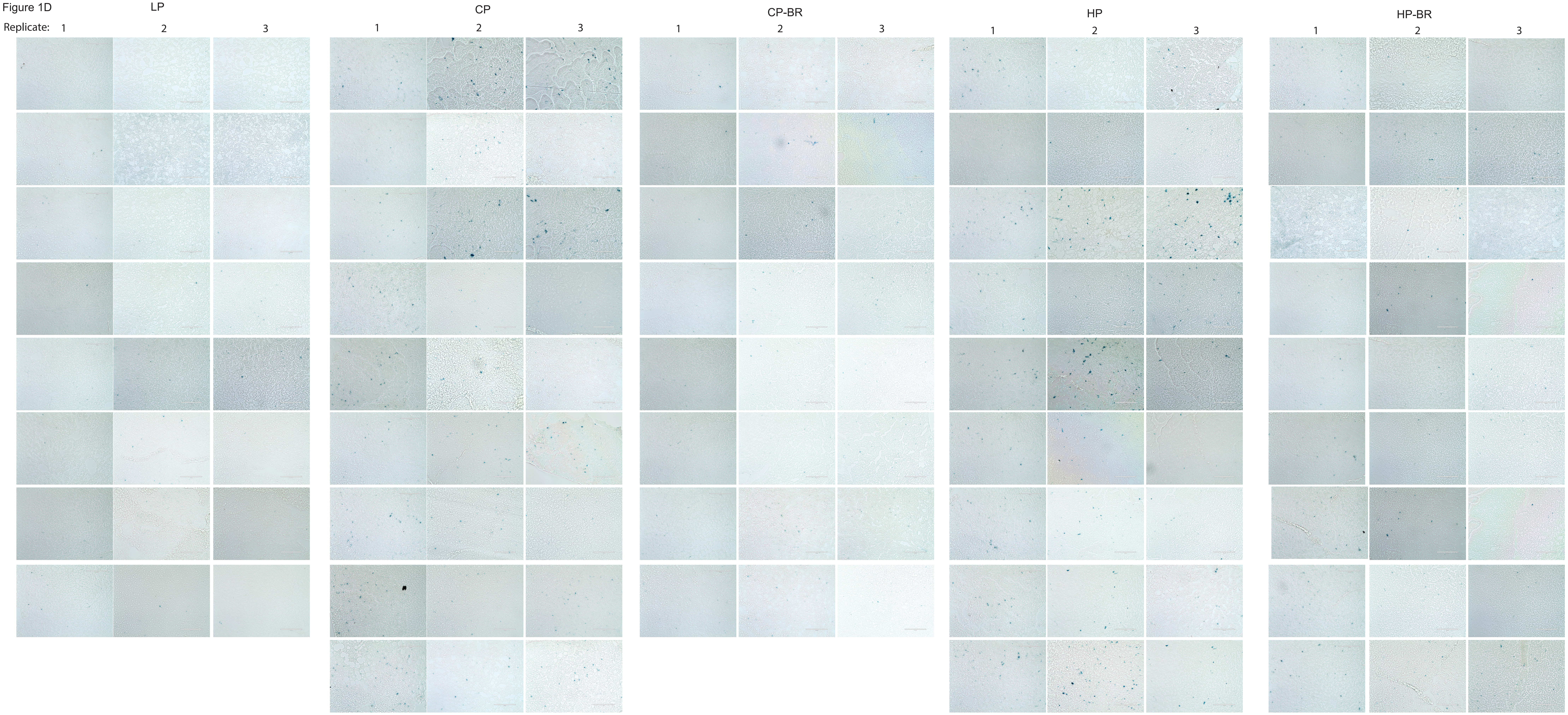

Figure 4G

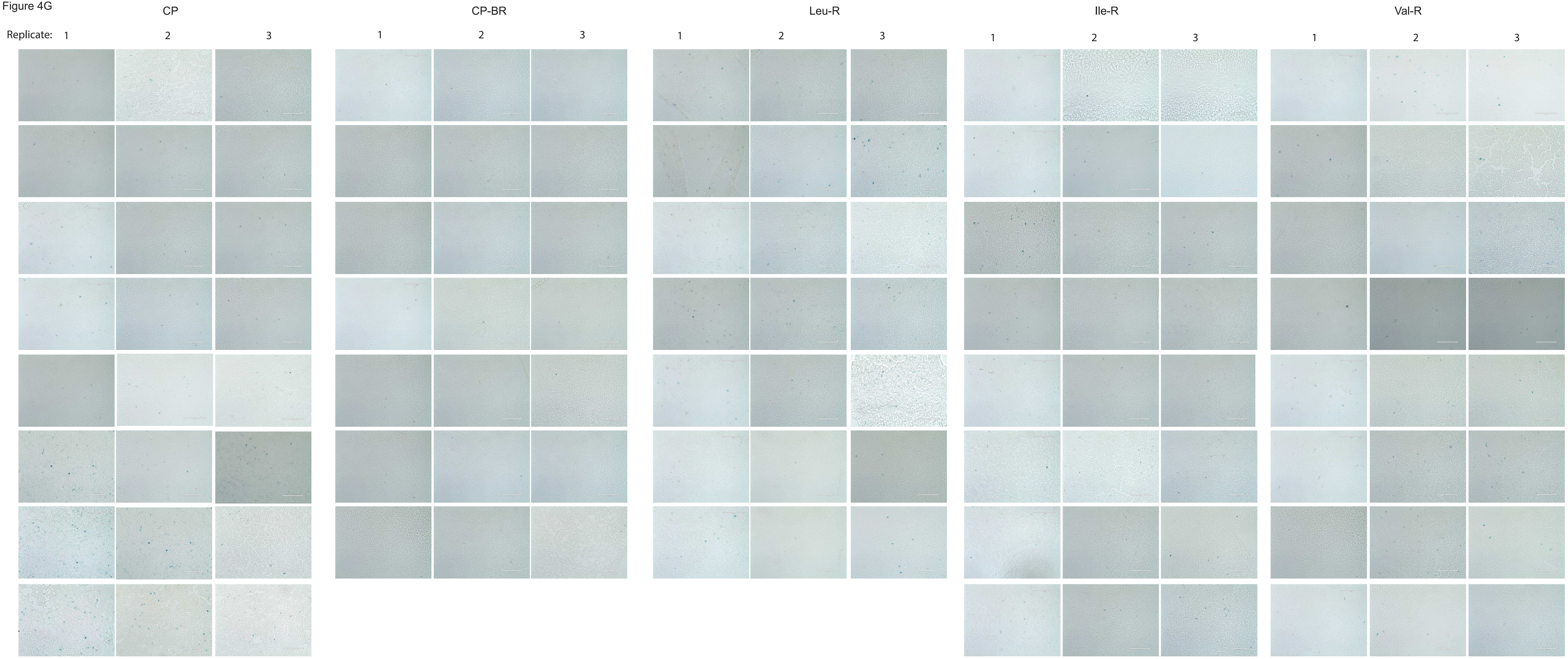

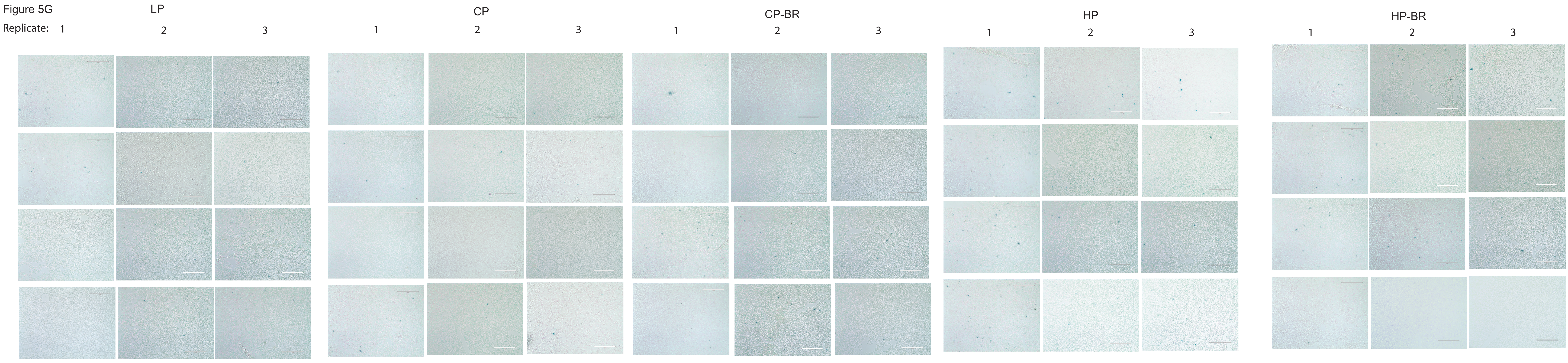
Figure 5G
Replicate:
LP
1 2 3
CP
1 2 3
CP-BR
1 2 3
HP
1 2 3
HP-BR
1 2 3

Supplementary Figure S7E-G

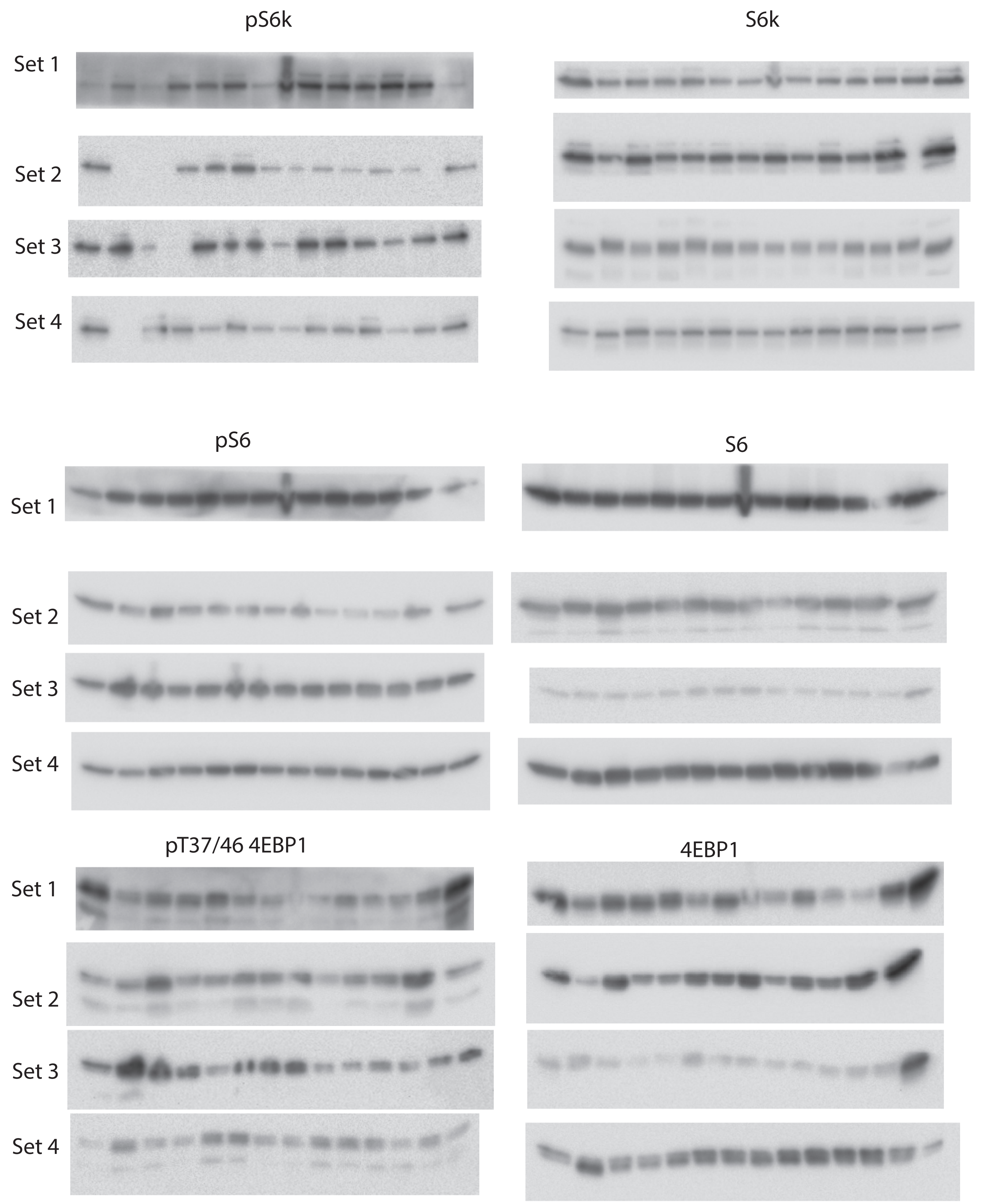

Supplementary Figure S7H-J

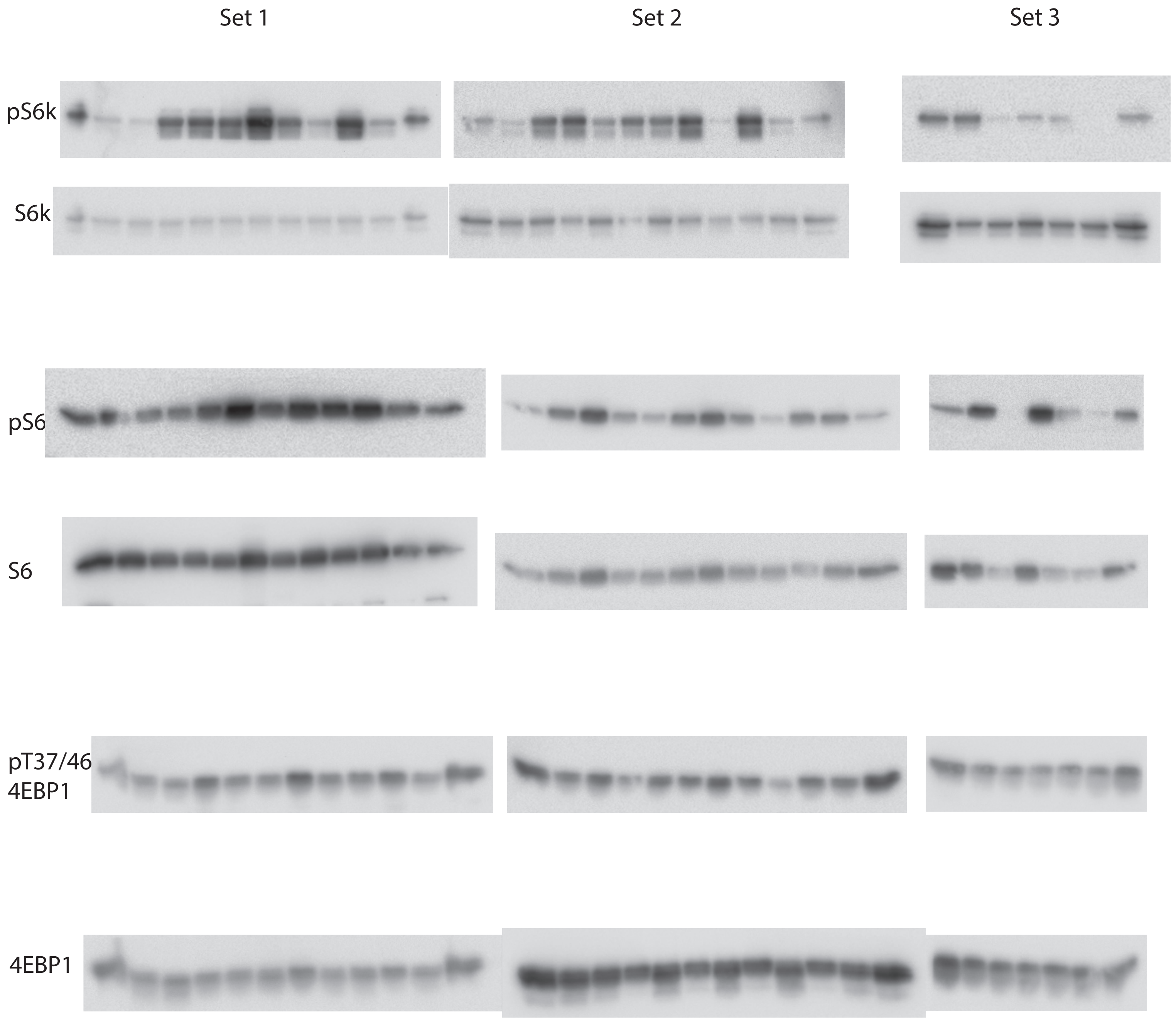

Supplement: Supplementary file 2 — Data S2. Source Images for Senescence‐Associated β‐Galactosidase Staining and Western blots. [file ACEL-24-e70176-s001.pdf]
