# Supplementary figures and images for: Tissue‐Specific Effects of Dietary Protein on Cellular Senescence Are Mediated by Branched‐Chain Amino Acids
Source: Aging Cell. 2025 Jul 28;24(10):e70176. doi: 10.1111/acel.70176 (PMC12507398; doi:10.1111/acel.70176)

Figure 3A - Liver 40X

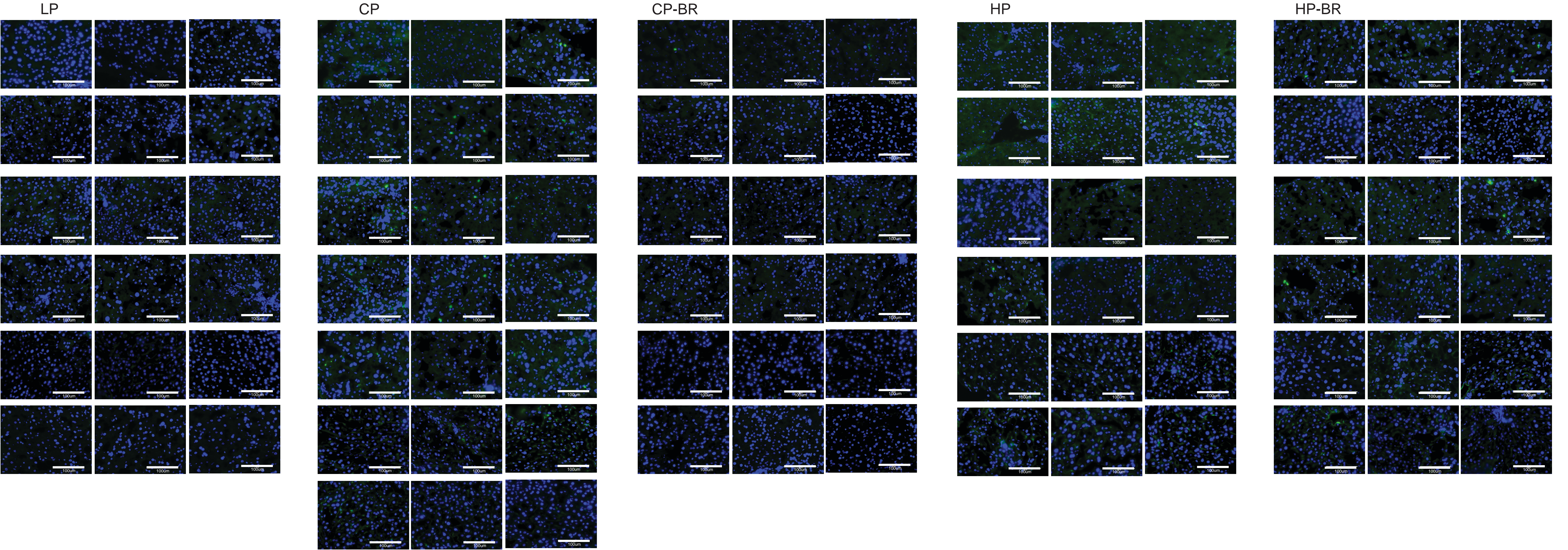

Supplement: Supplementary file 3 — Data S3. Source Images for gamma‐H2AX staining in Figure 3A. [file ACEL-24-e70176-s003.pdf]
